# Risk factors influencing chemotherapy compliance and survival of elderly patients with non-small cell lung cancer

**DOI:** 10.4314/ahs.v23i3.35

**Published:** 2023-09

**Authors:** Zhongxing Bing, Zhibo Zheng, Jiaqi Zhang

**Affiliations:** Department of Thoracic Surgery, Peking Union Medical College Hospital, Beijing 100005, China

**Keywords:** Chemotherapy compliance, elderly, non-small cell lung cancer, risk factor, survival analysis

## Abstract

**Objective:**

Non-small cell lung cancer (NSCLC) has high degree of malignance and proneness to recurrence and metastasis. The aim of this study was to analyse the risk factors influencing the chemotherapy compliance and survival status of elderly NSCLC patients.

**Methods:**

The clinical data of 110 patients admitted from January 2014 to March 2018 were retrospectively analysed. They were assigned to non-chemotherapy (n=25), partial chemotherapy (n=30) and complete chemotherapy (n=55) groups according to chemotherapy compliance, and followed up until March 2021. Their clinicopathological characteristics were investigated by univariate analysis and then multivariate Cox regression analysis. The survival rates were compared by Kaplan-Meier survival curve and log-rank test.

**Results:**

Among the 110 NSCLC patients, 25 did not receive chemotherapy, 30 underwent partial chemotherapy and 55 received complete chemotherapy. Educational level, pathological tumor-node-metastasis (TNM) stage, pathological type, surgical approach, place of residence, payment mode and chemotherapy stage were independent risk factors influencing the chemotherapy compliance (P<0.05).

**Conclusion:**

Particular attention should be paid to improving the chemotherapy compliance of patients with low educational level, late TNM stage, medical history of squamous cell carcinoma, history of thoracotomy, living in rural areas and no medical insurance, and those in the recurrence period or consolidation period of chemotherapy.

## Introduction

Non-small cell lung cancer (NSCLC) is a dominant type of primary lung cancer with high degree of malignance and proneness to recurrence and metastasis. The patients who suffer from NSCLC account for about 85% of all lung cancer cases^1^,^2^. According to the data in 2020, nearly 1.8 million people died of lung cance^3^, because most NSCLC patients have entered the advanced stage when diagnosed, for whom surgery no longer works. Although systemic chemotherapy can prolong patients' life, the treatment cycle is long and toxic side effects exist^4^. Additionally, poor compliance may be attributed to cancer-related fatigue and lack of perception and control for disease^5^. At present, related studies remain lacking.

Therefore, we herein retrospectively analysed the risk factors influencing chemotherapy compliance, and explored the survival status in 110 elderly patients with NSCLC, aiming to provide references for improving the prognosis.

## Materials and Methods

### Clinical data

The clinical data of 110 elderly patients with NSCLC admitted to our hospital from January 2014 to March 2018 were collected for retrospective analysis. They were aged 65-75 years old, (70.28±3.52) years old on average. According to chemotherapy compliance, the patients were assigned to non-chemotherapy (n=25), partial chemotherapy (n=30) and complete chemotherapy (n=55) groups. Partial chemotherapy meant that chemotherapy did not reach a course of treatment, while complete chemotherapy represented the completion of one course. Three consecutive weeks of chemotherapy were defined as a cycle, and four cycles were considered as a course of treatment. The follow-up ended in March 2021. This study was approved by the medical ethics committee of our hospital and informed consent was obtained from all patients and their family members.

The inclusion criteria were as follows: i) patients pathologically diagnosed as NSCLC after surgery, ii) those with requirement of paclitaxel/docetaxel/gemcitabine plus cisplatin (hereinafter termed chemotherapy) and who received entire treatment in our hospital, iii) those without other fatal diseases, and iv) those with complete clinical data, pathological information and follow-up data.

The exclusion criteria included: i) elderly patients without diagnosis or histopathological evidence of NSCLC, ii) those with a history of malignancy, iii) those who received surgery, radiotherapy or chemotherapy before, or iv) those with allergy to chemotherapy drugs or who lost to follow-up. The pathological stages of patients were classified strictly in accordance with the 7th edition of the American Joint Committee on Cancer (AJCC) tumor-node-metastasis (TNM) staging system for lung cancer. T classes (T1-T4) represented tumor size and local infiltration, N classes (N0-N3, N0: no lymph node involvement) denoted lymph node involvement, and M classes (M0 and M1) indicated distant metastasis^6^.

### Therapeutic protocols

Patients were intravenously dripped with 25 mg/m^2^ cisplatin (specification: 20 mg; Qilu Pharmaceutical Co., Ltd., China; batch No. 20121216] added in 500 mL of normal saline within 3 days before chemotherapy, and intravenously dripped with 35 mg/m^2^ docetaxel (specification: 10 mg; Hainan Sinochem Joint Pharmaceutical Industry Co., Ltd., China; H20057065)/100 mg/m2 gemcitabine (specification: 2,000 mg; Jiangsu Haosen Pharmaceutical Group Co., Ltd., China; H20030104), 1 h each time, on the 1st, 8th and 15th days. Slow intravenous bolus injection of 3 mg of granisetron (Sinopharm Group Guorui Pharmaceutical Co., Ltd., China; H20041206) was performed prior to each initiation of chemotherapy to prevent vomiting. Twenty-one days of chemotherapy were used as a cycle, and 4 cycles of chemotherapy were considered as a course of treatment.

### Collection of clinical data

The gender, age, smoking history, drinking history, tumor stage, family history and personal history of patients were collected.

### Follow-up

Follow-up was performed for all patients by telephone, outpatient visit and hospitalization, once every 3 months. The follow-up was ended in March 2021. The survival status, time of diagnosis, time of death or last follow-up and overall survival (OS) were recorded in details. OS was defined as the period from diagnosis to death or last follow-up.

### Statistical analysis

SPSS 21.0 software (IBM Inc., USA) was employed for statistical analysis. Measurement data were expressed as mean ± standard deviation (^-^x ± s), and the t-test was used for comparison between two groups. Count data were expressed as percentage (%) and compared using the χ^2^ test. The survival curve was plotted using the Kaplan-Meier method, and the survival rates were compared by the log-rank test. The Cox proportional hazards model was utilized to investigate the relationships between OS and clinical characteristics, and hazard ratios (HR) and corresponding 95% confidence interval (CI) were obtained from multivariate analysis. The clinical characteristics related to OS were employed to construct a nomogram model. P<0.05 represented statistically significant differences.

## Results

### Clinical characteristics of elderly patients with - NSCLC

The three groups had significantly different gender, long-term smoking history, educational level, Charlson comorbidity index, TNM stage, pathological type, surgical approach, place of residence, payment mode, chemotherapy stage, leukocyte count and neutrophils (P<0.05), but similar other baseline data and biochemical indicators (P>0.05) (Table 1).

**Table 1:** Clinical characteristics of elderly patients with NSCLC

Factor	Non-chemotherapy (n=25)	Partial chemotherapy (n=30)	Complete chemotherapy (n=55)	Statistical value	P
Gender				9.244	0.001
Male	15	6	21		
Female	10	24	34		
BMI				1.286	0.526
≥25 (kg/m^2^)	11	9	22		
<25 (kg/m^2^)	14	21	33		
Long-term drinking history	18	14	29	3.874	0.144
Long-term smoking history	19	20	26	6.847	0.033
Educational level				8.325	0.016
Junior high school and below	12	10	36		
Senior high school and above	13	20	19		
Charlson comorbidity index				13.868	0.031
0	1	0	6		
1	6	12	25		
2	8	14	15		
3	10	4	9		
TNM stage	17	23	27	6.883	0.032
Pathological type				10.910	0.028
Adenocarcinoma	5	14	30		
Squamous cell carcinoma	12	10	20		
Else	8	6	5		
Surgical approach				8.525	0.014
Thoracoscopy	21	14	31		
Thoracotomy	4	16	24		
Place of residence				13.046	0.001
Rural areas	19	25	26		
City and town	6	5	29		
Payment mode				6.780	0.034
Medical insurance	13	13	39		
Non-medical insurance	12	17	16		
Chemotherapy stage				13.451	0.009
Induced remission period	15	10	13		
Consolidation treatment period	6	12	16		
Refractory recurrence period	4	8	26		
Leukocyte count (×10^9^/L)	4.02±1.56	4.16±1.06	4.51±1.02	10.344	<0.001
Erythrocyte count (×10^12^/L)	5.96±1.06	5.54±0.92	5.25±0.56	2.216	0.058
Platelets (×10^9^/L)	190.25±44.56	205.09±40.32	209.35±41.06	1.805	0.073
Hemoglobin (g/L)	130.36±5.25	137.96±4.79	142.93±6.37	0.864	0.389
Neutrophils (×10^9^/L)	3.36±0.87	3.18±0.52	3.07±0.59	9.726	<0.001
Lymphocytes (×10^9^/L)	1.62±0.56	1.79±0.59	3.01±1.02	1.592	0.057
Monocytes (×10^9^/L)	0.24±0.05	0.25±0.06	0.32±0.59	1.335	0.092

### Multivariate Cox regression analysis results of influencing factors of chemotherapy compliance and receiver operating characteristic (ROC) curve

**Figure 1:**
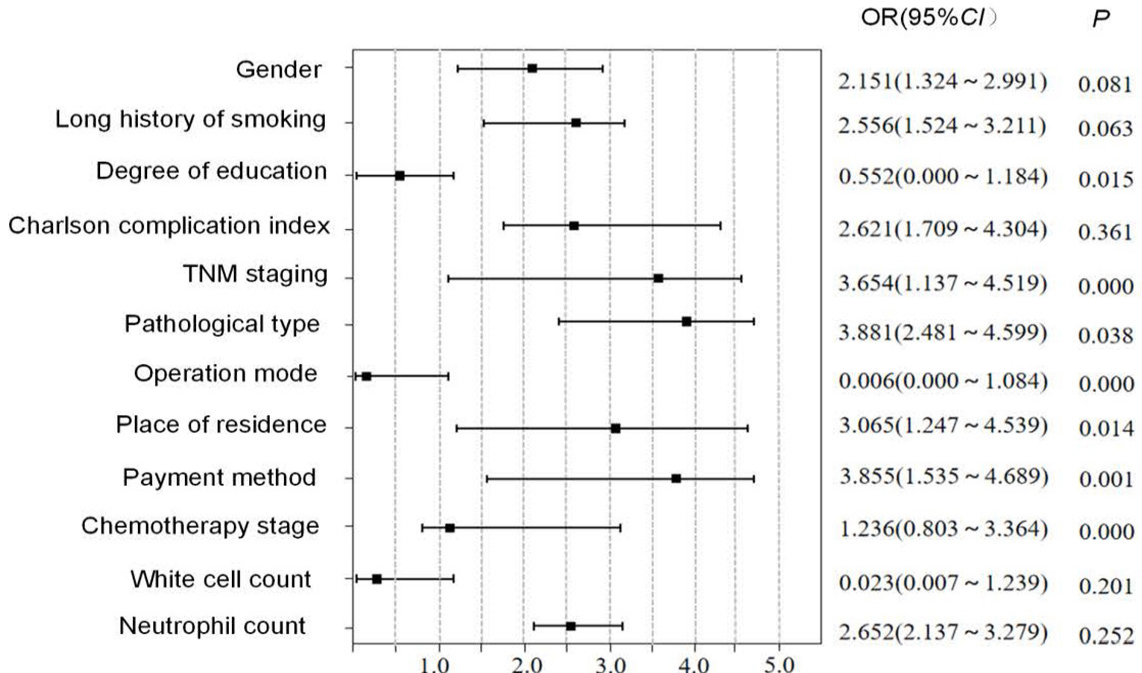
Multivariate Cox regression analysis results of chemotherapy compliance in elderly patients with NSCLC

**Figure 2:**
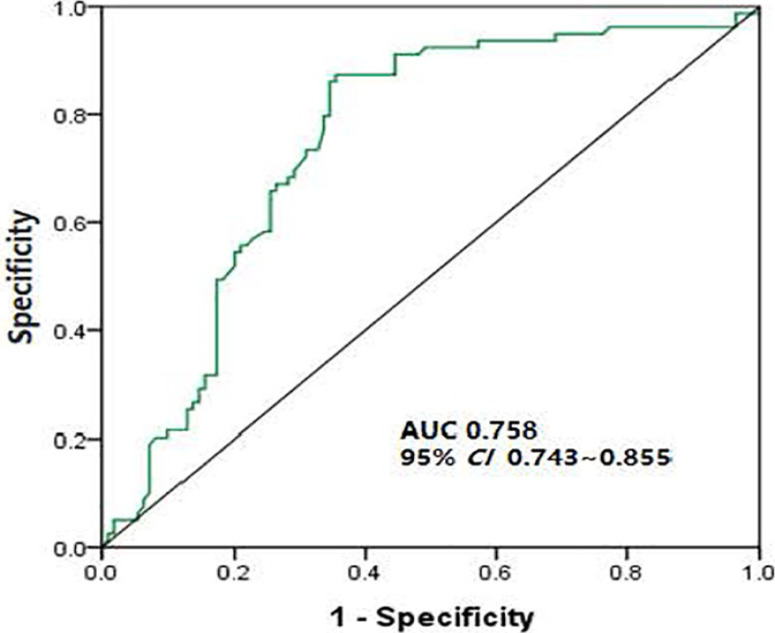
ROC curves for risk factors influencing chemotherapy compliance and survival of elderly patients with NSCLC

The categorical variables that influenced the chemotherapy compliance of elderly patients with NSCLC were assigned (Table 2). Chemotherapy compliance was selected as a dependent variable, and variables with statistical significance in the chi-square test and one-way ANOVA as independent variables were incorporated into multivariate Cox regression analysis. The results revealed that educational level, TNM stage, pathological type, surgical approach, place of residence, payment mode and chemotherapy stage were independent risk factors influencing the chemotherapy compliance of elderly patients with NSCLC (P<0.05) (Figure 1). The area under the ROC curve was 0.758 (95% CI: 0.743-0.855), indicating that the model had high predictive value (Figure 2).

**Table 2:** Assignment of categorical variables

Variable	Assignment
Gender	Male=1, female=0
BMI	≥25 kg/m^2^=1, <25 kg/m^2^=0
Long-term drinking history	Yes=1, no=0
Long-term smoking history	Yes=1, no=0
Educational level	Junior high school and below=1, senior high school and above=2
TNM stage	Stage I=1, stage II=2, stage III=3
Pathological type	Adenocarcinoma=1, squamous cell carcinoma=2, others=3
Surgical approach	Thoracoscopy=1, thoracotomy=2
Place of residence	Rural areas=1, city and town=2
Payment mode	Non-medical insurance=1, medical insurance=2
Chemotherapy stage	Induced remission period=1, consolidation treatment period=2, refractory recurrence period=3

Charlson comorbidity index, leukocyte count, erythrocyte count, platelets, hemoglobin, neutrophils, lymphocytes and monocytes were input in light of the original data.

### Variable collinearity diagnosis results

The screened variables were subjected to collinearity diagnosis analysis. The variance inflation factor was lower than 10, suggesting that the variables were independent without collinearity (Table 3).

**Table 3:** Collinearity diagnostic coefficients of variables

Variable	Tolerance	Variance inflation factor
Educational level	0.945	2.013
TNM stage	0.946	3.291
Pathological type	0.917	3.022
Surgical approach	0.963	1.045
Place of residence	0.925	6.765
Payment mode	0.913	3.654
Chemotherapy stage	0.962	4.365

### Sensitivity to chemotherapeutic drugs in elderly patients with NSCLC

The results of collagen gel droplet culture drug-sensitivity test were obtained from 110 elderly patients with NSCLC, and then a drug to which cancer cells were most sensitive was screened, i.e., the drug can kill most cancer cells in vitro. The sensitivities of three chemotherapeutic drugs used in this study followed a descending order of gemcitabine + cisplatin (40.00%, 44/110), docetaxel + cisplatin (32.73%, 36/110), and paclitaxel + cisplatin (29.09%, 30/110). In the detection process, however, the cancer cells in some patients remained insensitive (Table 4).

**Table 4:** Sensitivity to three chemotherapeutic drugs

Variable	Sensitive	Insensitive	Sensitivity rate (%)
Gemcitabine	44	66	40.00
Docetaxel	36	74	32.73
Paclitaxel	30	80	29.09

### Survival analysis results of chemotherapy in elderly patients with NSCLC

**Figure 3:**
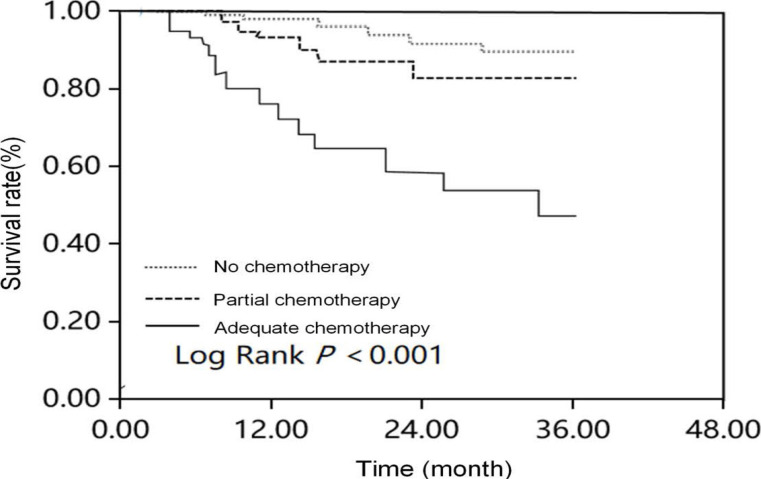
Influence of chemotherapy compliance on survival rate in elderly patients with NSCLC

The median survival time periods of non-chemotherapy, partial chemotherapy and complete chemotherapy groups were 20, 24 and 36 months, respectively. Moreover, the influence of chemotherapy compliance as a grouping factor on the survival rate of elderly patients with NSCLC was analysed, suggesting a significant difference (P<0.001) (Figure 3).

### Construction of nomogram prediction model for risk factors influencing chemotherapy compliance and survival

**Figure 4:**
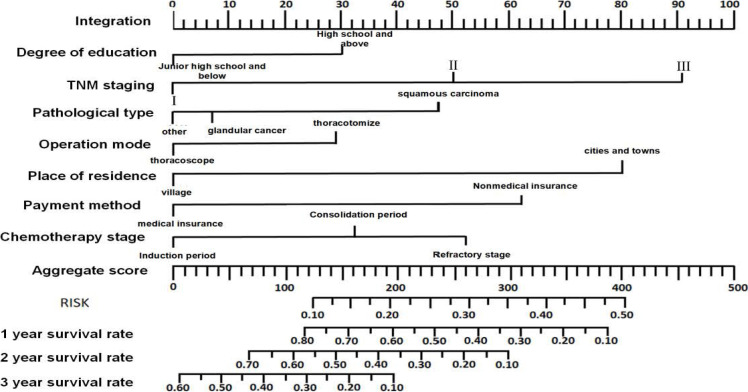
Nomogram model for risk factors influencing chemotherapy compliance and survival

The independent predictive factors obtained from multivariate Cox regression analysis were selected as predictors to construct a nomogram model for predicting the risk factors influencing the chemotherapy compliance and probability of survival of elderly patients with NSCLC (Figure 4). Firstly, the value of each variable was positioned on the item scale, and then a vertical line on the individual score axis in the first row corresponding to the value point of each variable was drawn to identify the score. The sum of the individual scores of all variables represented the total score of chemotherapy compliance, and the total score vertically corresponded to the probability of occurrence. The results exhibited that the predictive ability of TNM stage was highest, followed by those of place of residence and then educational level.

### Accuracy evaluation results of nomogram model

**Figure 5:**
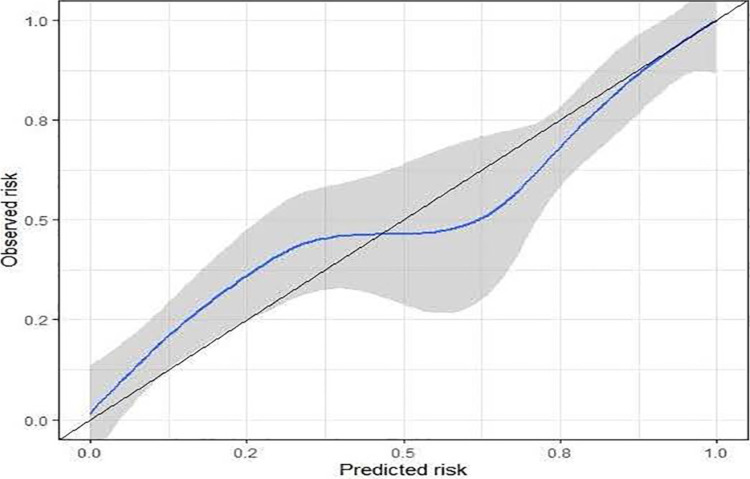
Calibration curve of prediction model for risk factors influencing chemotherapy compliance and survival

The calibration curve of the prediction model was plotted (Figure 5). The gray diagonal was the reference line, while the blue curve was the fitting line, and the gray shadow was 95% CI. When the event rate was higher than 45%, the model overestimated the risk. When the event rate was below 45%, the model underestimated the risk. Overall, this model had high accuracy.

### Consistency evaluation results of nomogram model

**Figure 6:**
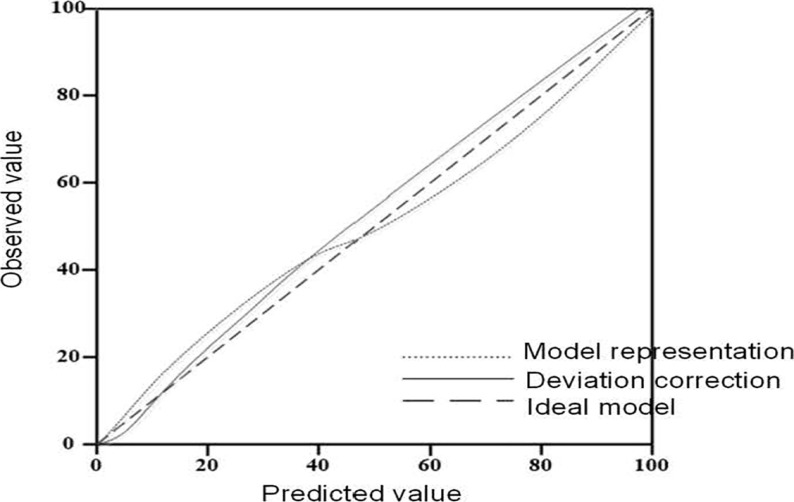
Calibration curve of nomogram model for risk factors influencing chemotherapy compliance and survival

The consistency test was conducted by plotting a calibration curve between the predicted and actual values of the prediction model. The actual curve in the calibration curve was well fitted with the ideal curve, indicating that the prediction using the nomogram model for risk factors was consistent with the actual situation (Figure 6).

## Discussion

Lung cancer is one of the most common malignancies worldwide, with the highest mortality rate among those of all malignancies in China^7^. Surgery has been proven to be one of the indispensable treatment methods for NSCLC^8^. However, most patients have been in late stage when diagnosed, especially in the elderly. Thus, chemotherapy may be beneficial to the elderly with organ degenerative changes and reductive metabolism and protein binding. Paclitaxel is a novel anti-microvascular agent that can kill tumor cells without affecting immune function, and its combination with cisplatin can maximize the drug efficacy^9^. As a drug designed and synthesized based on paclitaxel with a similar mechanism, docetaxel is capable of promoting tubulin polymerization, thus suppressing cell mitosis in a stable manner^10^. Gemcitabine is a synthetic pyrimidine nucleotide drug that can markedly destroy DNA of cancer cells^11^. Consequently, paclitaxel/docetaxel/gemcitabine plus cisplatin is commonly used to treat NSCLC in clinical practice.

In the present study, chemotherapy with paclitaxel/docetaxel/gemcitabine plus cisplatin was conducted for 110 elderly patients with NSCLC. Among them, 25 patients (22.73%) did not receive chemotherapy, 30 patients (27.27%) underwent partial chemotherapy and 55 patients (50.00%) received complete chemotherapy. Multivariate Cox regression analysis revealed that TNM stage and chemotherapy stage were independent risk factors that influenced their chemotherapy compliance, being consistent with the results reported by Wang and Licht *et al.*^12^,^13^ Probably, accurately determining the TNM stage is conducive to the formulation of appropriate therapeutic protocols. Considering that tumor size is small and lymph node metastasis is unobvious in the early stage of NSCLC, early chemotherapy can raise the survival rate and improve the quality of life.

NSCLC patients pathologically diagnosed as adenocarcinoma have good chemotherapy compliance^14^. Likewise, we herein found that the numbers of patients receiving complete chemotherapy, partial chemotherapy and non-chemotherapy were 16 vs. 14, 12 vs. 10, and 7 vs. 8 respectively between adenocarcinoma and squamous cell carcinoma, with significant differences (P<0.05). Possibly, some patients with squamous cell carcinoma lost the opportunity of radical surgery due to the difficulty of operation, while the patients diagnosed as adenocarcinoma had lower degree of malignance and treatment difficulty and better prognosis, which increased their confidence and thus augmented the chemotherapy compliance^15^,^16^. Moreover, patient's educational level, place of residence and payment mode are also independent risk factors influencing the chemotherapy compliance^14^, which have been confirmed in this study. The patients who have higher educational levels may better understand the therapeutic protocols. Besides, the treatment cost of cancer is generally high at present, and the economic burden on patients' family members increases in the absence of medical insurance, so they no longer afford chemotherapy^14^,^17^. In addition, the surgical approach also affects the chemotherapy compliance of elderly patients with NSCLC. Generally, postoperative adjuvant chemotherapy is needed for patients who receive surgical treatment^18^.

In this study, thoracoscopy was superior to thoracotomy for radical resection with respect to postoperative adjuvant chemotherapy compliance, being consistent with the study of Petersen et al.^19^ Probably, conventional adjuvant chemotherapy was started later after thoracotomy than thoracoscopy, so the chemotherapy dose was lower^20^, ^21^.

In this study, the patients were followed up for three years, and 45 died. There were significant differences between the survival rates of non-chemotherapy and complete chemotherapy groups as well as partial chemotherapy and complete chemotherapy groups, but non-chemotherapy and partial chemotherapy groups had similar survival rates. Accordingly, enhancing the chemotherapy compliance of elderly patients with NSCLC can elevate the survival rate.

In conclusion, educational level, TNM stage, pathological type, surgical approach, place of residence, payment mode and chemotherapy stage are independent risk factors that influence the chemotherapy compliance of elderly patients with NSCLC. In addition, complete chemotherapy can improve the survival rate of patients. Therefore, interventions should be targeted toward elderly NSCLC patients concerning the educational level, TNM stage, pathological type, surgical approach, place of residence, payment mode and chemotherapy stage, thus enhancing the chemotherapy compliance and improving the prognosis. Regardless, this study is limited. First, this is a retrospective study. Second, the sample size is small. Hence, further prospective studies with larger sample sizes are still in need to validate the findings herein.
